# An abundant bacterial phylum with nitrite-oxidizing potential in oligotrophic marine sediments

**DOI:** 10.1038/s42003-024-06136-2

**Published:** 2024-04-11

**Authors:** Rui Zhao, Steffen L. Jørgensen, Andrew R. Babbin

**Affiliations:** 1 Department of Earth, Atmospheric and Planetary Sciences, Massachusetts Institute of Technology, Cambridge, MA USA; 2 Centre for Deep-Sea Research, Department of Earth Science, University of Bergen, Bergen, Norway

**Keywords:** Element cycles, Water microbiology

## Abstract

Nitrite-oxidizing bacteria (NOB) are important nitrifiers whose activity regulates the availability of nitrite and dictates the magnitude of nitrogen loss in ecosystems. In oxic marine sediments, ammonia-oxidizing archaea (AOA) and NOB together catalyze the oxidation of ammonium to nitrate, but the abundance ratios of AOA to canonical NOB in some cores are significantly higher than the theoretical ratio range predicted from physiological traits of AOA and NOB characterized under realistic ocean conditions, indicating that some NOBs are yet to be discovered. Here we report a bacterial phylum *Candidatus* Nitrosediminicolota, members of which are more abundant than canonical NOBs and are widespread across global oligotrophic sediments. *Ca*. Nitrosediminicolota members have the functional potential to oxidize nitrite, in addition to other accessory functions such as urea hydrolysis and thiosulfate reduction. While one recovered species (*Ca*. Nitrosediminicola aerophilus) is generally confined within the oxic zone, another (*Ca*. Nitrosediminicola anaerotolerans) additionally appears in anoxic sediments. Counting *Ca*. Nitrosediminicolota as a nitrite-oxidizer helps to resolve the apparent abundance imbalance between AOA and NOB in oxic marine sediments, and thus its activity may exert controls on the nitrite budget.

## Abstract (web summary)

A novel phylum of bacteria widespread in oligotrophic marine sediments are capable of oxidizing nitrite to nitrate and could resolve the apparent abundance mismatch between ammonia- and nitrite-oxidizers.

## Introduction

Nitrite is an important intermediate compound in the biogeochemical nitrogen cycle, whose cycling dictates the availability of fixed nitrogen in marine ecosystems. Nitrite is controlled by multiple metabolic pathways: it can be produced by nitrate reduction and aerobic ammonia oxidation, and consumed by nitrite reduction and nitrite oxidation^1^. Among these pathways, by converting nitrite to nitrate, nitrite oxidation is a critical control point retaining bio-available nitrogen in an ecosystem by limiting the further reduction of nitrite to nitrogen gas^2^. Nitrite oxidation is mediated by a phylogenetically diverse functional guild known as the nitrite-oxidizing bacteria (NOB), which has been studied in a range of ecosystems, such as engineered environments^3,4^, coastal sediments^5–7^, haloalkaline lake sediments^8^, hot springs^9–11^, seawater^12,13^, and oxygen deficient zones^14,15^. However, the diversity and metabolic capacities of NOB in deep-sea sediments have not been well studied.

Nitrification is catalyzed by two different chemolithoautotrophic guilds, ammonia oxidizers and nitrite oxidizers, and is an important nitrogen cycling process in global marine sediments. Nitrifiers are one of the most dominant (typically > 10%) functional guilds among the microbial taxa in oxic sediments^16,17^, which account for a considerable proportion of the global seafloor^18^. Nitrite rarely accumulates in such sediments^19^, where newly produced nitrite is rapidly oxidized to nitrate by NOB due to the presence of oxygen. The absence of appreciable nitrite (and ammonium) in this zone also indicates that NOBs are as efficient in the oxidation of nitrite as ammonia-oxidizing archaea (AOA) in the oxidation of ammonium released from organic matter degradation, as has been observed in the dark ocean interior [e.g., see refs. ^20–22^].

For the process of ammonia oxidation in marine sediments, AOA are well known to dominate over ammonia-oxidizing bacteria^16,23,24^ and numerous studies have quantified their activity, regulation, power requirement, and genetic identity^17,25–28^. By comparison, our knowledge about microorganisms involved in nitrite oxidation in marine sediments is extremely limited. Previously, gene-based surveys have indicated the presence of *Nitrospinaceae* and *Nitrospiraceae* in marine sediments^16,24,29^, with a few cultured representatives from coastal sediments^5–7^. However, it remains unclear whether members of *Nitrospinaceae* and *Nitrospiraceae* (i.e., the canonical marine nitrite oxidizers) are in fact the major NOBs in marine sediments. Importantly, NOB abundances in some deep-sea sediments have been observed to be orders of magnitude lower than those of AOA^16,24^. This perhaps indicates that the majority of NOB in this vast habitat have not been identified, yet limited case studies exist. If, however, AOA severely outnumber NOB in oxic marine sediments, the nitrogen cycle would not be closed without a cryptic nitrite loss process, presumably denitrification, that consumes bio-available nitrogen rather than recycling it^2^.

In this study, we first highlight an abundance mismatch between AOA and canonical NOB in some oxic sediments, based on a compilation of quantitative data in 16 marine sediment cores. To address this discrepancy, we rely on metagenome sequencing data from the Arctic Mid-Ocean Ridge to discover overlooked NOBs. We focus on three metagenome-assembled genomes (MAGs) that contain the metabolic potential of nitrite oxidation and form a bacterial phylum different from previously known NOB phyla. We then search for the presence of these novel NOB across global marine sediments. We conclude by calculating the abundance ratio of AOA to NOB including this more abundant phylum to resolve the previously identified discrepancy.

## Results and discussion

### Abundance mismatch between AOA and canonical NOB in some oxic deep-sea sediments

In order to investigate the reasons for the often-observed offset between the abundances of AOA and NOB, we explored the theoretical abundance ratio between them based on their (i) biomass yields (carbon synthesized per nitrogen oxidized) and (ii) cell quotas (mass of carbon per cell), and also considered (iii) mortality/grazing rates and (iv) relevant environmental conditions. Because AOA and NOB in deep-sea sediments are mostly uncultured, we performed the simple calculation based on the physiological traits of AOA and NOB isolates grown under relevant oceanic conditions^30^. Marine AOA exhibit approximately 1.6–3.1 times (mean value 2.3) higher biomass yields than NOB but maintain only 0.20–0.63 (mean value 0.30) times the cell quota of NOB^30^. In oxic environments without significant nitrite accumulation, because of the balanced bulk reaction rates of ammonia and nitrite oxidation, the cell abundance of AOA should be theoretically ~6.9 times (range: 2.6–15.8) higher than that of NOB. The difference in mortality/loss rates^20,31^ caused by grazing and/or viral lysis has been invoked to explain the relative abundance difference between AOA and NOB in the ocean, although the recent redox-based mechanistic model of Zakem et al.^32^ suggests that this is not necessary. Because of the assumed equivalent rates of the tightly coupled ammonia and nitrite oxidation processes, this theoretical ratio is likely suitable only for predicting the AOA:NOB abundance ratio in well-oxygenated environments without nitrite accumulation, such as oxic sediments and the ocean interior^32^. Indeed, the 7- to 11-fold abundance differences between AOA and NOB observed in a recent deep ocean compilation^32^ fall within this theoretical ratio derived from growth characteristic observations. While AOA and NOB are found in some anoxic [e.g., refs. ^31,33–35^] or fluctuating (e.g., with diel/seasonal variations) environments, under such conditions their metabolic activities are not necessarily coupled. In anoxic settings, other metabolisms like denitrification can supply and consume nitrite and nitrifiers may engage in other metabolisms^36,37^, and thus the relative abundances of AOA and NOB may not be tightly correlated.

**Fig. 1:**
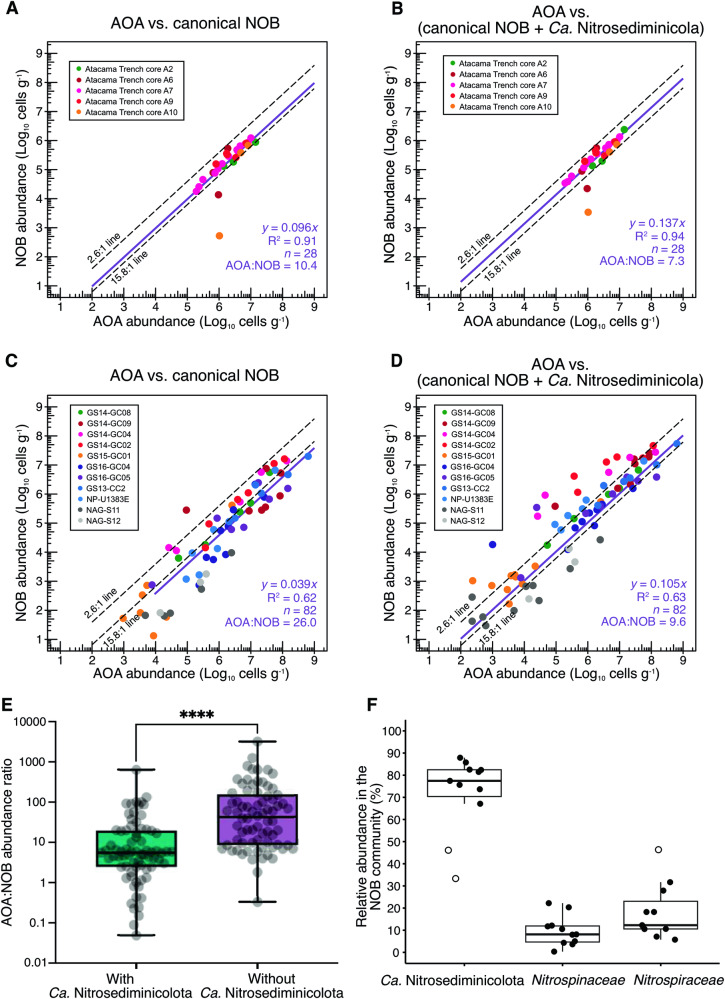
Comparison of the abundances of ammonia-oxidizing archaea (AOA) and nitrite-oxidizing bacteria (NOB) in oxic marine sediments. **A** Abundances of AOA and canonical NOB (affiliated with *Nitrospiraceae* and *Nitrospinaceae*) in a total of 28 samples of five sediment cores with extensive oxic zones in the Atacama Trench. **B** Same as (**A**), but AOA vs NOB including *Candidatus* Nitrosediminicolota. **C** Abundances of AOA and canonical NOB (affiliated with *Nitrospiraceae* and *Nitrospinaceae*) in a total of 82 samples of 11 sediment cores with extensive oxic zones in the Arctic and Atlantic Oceans. **D** Same as (**C**), but AOA vs NOB including *Ca*. Nitrosediminicolota.  The best fit linear regressions (on raw, not log-transformed data) are included as solid purple lines with noted statistics. To facilitate the comparison, two dashed lines delineating the theoretical AOA:NOB abundance ratio range of 2.6–15.8 are included. **E** Abundance ratios of AOA to NOB with and without *Ca*. Nitrosediminicolota in the total 82 Arctic and Atlantic sediment samples. The median is noted by the black line and the colored boxes show the 99% confidence intervals. The whiskers denote the full range of observations. **F** Depth-integrated relative abundances of the three NOB lineages in the oxic zones of the 11 Arctic and Atlantic sediment cores. Boxes indicate 95% confidence intervals with the median displayed as a bold line. Outliers are marked with open circles.

We tested whether the theoretical AOA:NOB abundance ratio applies in oxic marine sediments. Restricting our analysis to oxic layers, we focused on a series of deep-sea (depth > 1000 m) sediment cores^16,17,38–40^, in which the thick oxic zones permitted high-resolution profiling of both geochemistry (especially oxygen) and microbial community composition. We compared the abundances of AOA and NOB, initially assuming that only members of the canonical marine NOB families *Nitrospinaceae* and *Nitrospiraceae* perform nitrite oxidation. In five sediment cores comprising 28 total depth layers from the Atacama Trench^40^ in the East Pacific Ocean (Supplementary Data 1), we observed a correlation between the abundances of AOA and NOB, with an AOA:NOB abundance ratio of 10.5 (*R*^2^ = 0.91, Fig. 1A). That this observed abundance ratio is within the theoretical range, canonical NOB families likely prevail in the Atacama Trench and novel nitrite oxidizers need not be invoked.

However, such an abundance match between AOA and NOB was not always observed in marine sediments. When extending the comparison to more diverse cores beyond deep trench systems (eight from the Arctic Mid-Ocean Ridge (AMOR) [four cores reported in Zhao et al.^38^, plus GS13-CC2^41^, GS14-GC04^19^, GS14-GC02, and GS15-GC01^39^], one piston core (NP-U1383E^16,28^) retrieved from the North Pond of the Mid-Atlantic Ridge, and two piston cores from the North Atlantic Gyre^17^) (Supplementary Data 1), we found that the NOB across these cores are far outnumbered by AOA beyond the upper theoretical limit at many investigated depths (Fig. S1). When combining observations from all 11 sediment cores (a total of 82 unique samples) (Fig. 1C), we observed that the abundances of AOA and NOB showed a linear relationship again, but with a slope of 26:1. The AOA:NOB abundance ratio is higher than the upper boundary (15.8) of the theoretical range in 56 of 82 samples (median = 43.3, with the 99% confidence interval [16.6, 83.9]; Fig. 1E). The apparent excess of AOA over NOB in these cores indicates that (i) some AOA are inactive or do not contribute to nitrite production, or more likely, (ii) there are yet unidentified NOBs present.

### A bacterial phylum *Candidatus* Nitrosediminicolota defined by MAGs from marine sediments

To elucidate which microbes are likely overlooked NOBs in marine sediments, we focused on the metagenome sequencing data generated for two investigated sediment locations: four sediment horizons of AMOR core GS14-GC08, four horizons of NP-U1383E of North Pond^38^. Through genome binning and refinement, we noticed three MAGs (Bin_086, Bin_096, and Bin_108) containing genes encoding the nitrite-oxidizing enzyme nitrite oxidoreductase (Nxr) but not affiliated with any well-defined bacterial phylum. All three MAGs are of high completeness ( > 92%; Table 1) and low fragmentation ( < 73 scaffolds; Table 1) and therefore should be regarded as high-quality genomes. The genome sizes are in the range of 1.8–2.4 Mbp. Automatic classification based on the 120 bacterial single-copy genes suggests that they are affiliated with an understudied bacteria phylum (with the placeholder JADFOP01 in the GTDB RS214 Release), which previously included three MAGs (B6D1T2, B58T1B8, and B13D1T1) recovered from hadal sediments beneath the Mariana Trench^42^.

**Table 1:** Genome quality of *Candidatus* Nitrosediminicolota genomes in marine sediments

	*Ca*. Nitrosediminicola aerophilus (Bin_096^a^)	*Ca*. Nitrosediminicola anaerotolerans (Bin_086^a^)	*Ca*. Nitrosediminicola anaerotolerans (Bin_108^a^)	B13D1T1^b^	B6D1T2^b^	B58T1B8^b^
Completeness^c^	92.8%	94.2%	94.2%	86.2%	97.1%	96.2%
Contamination^c^	0.83%	0.96%	0.42%	2.3%	0.9%	3.4%
Total length (base pairs)	1,837,265	2,183,767	2,454,095	1,632,813	2,151,810	2,516,409
GC content	60.6%	62.3%	62.3%	60.9%	59.9%	59.2%
Number of scaffolds	73	48	61	322	84	155
Number of contigs	78	62	63	325	89	160
N50 of contigs	63,404	116,403	61,632	5514	128,070	23,346
# coding sequences	1770	2075	2372	1679	2083	2440
Coding density	89.0%	86.9%	86.2%	88.7%	87.6%	85.9%
rRNA	0	6	2	0	3	3
tRNA	33	49	47	31	49	44

^a^MAGs recovered from Arctic Mid-Ocean Ridge sediments in this study.

^b^MAGs recovered from Mariana Trench sediments by ref. ^42^.

^c^Based on lineage-specific marker sets determined with CheckM2.

**Fig. 2:**
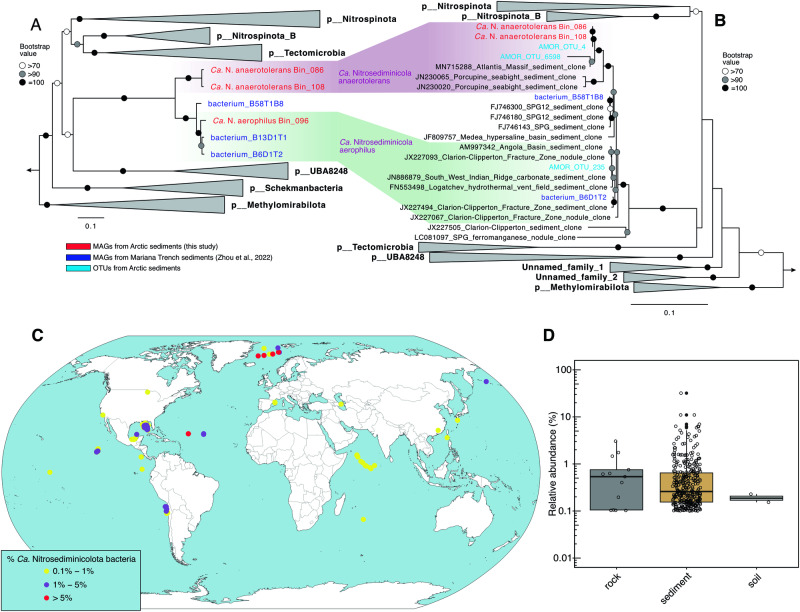
Phylogeny and distribution of *Candidatus* Nitrosediminicolota. **A** Maximum-likelihood phylogenetic tree of *Ca*. Nitrosediminicolota and related phyla based on the 16 S rRNA gene. **B** As (**A**), but the maximum-likelihood phylogenetic tree of *Ca*. Nitrosediminicolota and related phyla based on the concatenated 120 single-copy genes of bacteria. Both trees are rooted in five Methylamirales genomes. The three MAGs recovered from AMOR sediments are highlighted in red, the MAGs from the Mariana Trench in dark blue, and the OTUs from Arctic sediments in cyan. The nomenclature of the bacterial phyla follows GTDB, except that *Ca*. Nitrosediminicolota was proposed in this study. Bootstrap values of > 70 (*n* = 1000) are shown with symbols listed in the legend. The scale bars show estimated sequence substitutions per residue. **C** Global distribution of *Ca*. Nitrosediminicola bacteria. Except for two soil sites and two basaltic rock sites, *Ca*. Nitrosediminicola bacteria are present in multiple depths of each of the sediment cores represented by individual circles. In each core, the maximum relative abundance is shown using different colors as listed in the legend. The basal global map was created in R using free vector and roster map data from Nature Earth (https://www.naturalearthdata.com/). **D** Relative abundances of *Ca*. Nitrosediminicolota bacteria in three major habitats where they were detected at > 0.1% relative abundance.

The novel phylogenetic affiliation of the now total six MAGs included in the JADFOP01 phylum is confirmed by phylogenetic analyses. Within the phylogenetic trees based on the concatenated 120 bacterial single-copy genes (Fig. 2B) and 14 conservative single-copy ribosomal proteins (Fig. S2), the six MAGs form a branch separated from several established bacterial phyla such as Nitrospinota (containing NOB), Tectomicrobia, Nitrospinota_B, Schekmanbacteria, and UBA8248 (Fig. 2A). The average nucleotide identities (ANIs) between members of JADFOP01 and those in the established phyla are in the range of 47–53%, much lower than the threshold of 83% distinguishing bacterial phyla^43^ and supporting the view that these MAGs represent a distinct bacterial phylum. The novel phylogenetic affiliations of these MAGs are confirmed by phylogenetic analysis based on the 16 S rRNA gene, which shows a congruent topology with that based on both 120 single-copy genes and ribosomal proteins (Fig. 2B). We tentatively name this phylum *Candidatus* Nitrosediminicolota, for their prevalence in globally distributed marine sediments (see Etymology description).

The calculated average amino acid identities (AAIs) among the six MAGs are >80% (Fig. S3), placing them in the range suggested for genomes belonging to the same genus [65–95%^43^]. We tentatively name this genus *Candidatus* Nitrosediminicola. Within this genus, three MAGs (Bin_096, B6D1T2, and B13D1T1) show AAIs higher than 95% and should fall into the same species, for which we suggest a provisional name *Candidatus* Nitrosediminicola aerophilus. Bin_086 and Bin_108 also shared an AAI higher than 95% and belong to the same species, which we provisionally name *Candidatus* Nitrosediminicola anaerotolerans. The remaining MAG B58T1B8 shows AAIs <79% with all other *Ca*. Nitrosediminicola MAGs and therefore should represent a third species that we do not name as it is not found in our samples. Therefore, the six MAGs of *Ca*. Nitrosediminicola reported here should be resolved to three species within a single genus.

### *Ca*. Nitrosediminicolota is prevalent in oligotrophic marine sediments

To explore the global occurrence of *Ca*. Nitrosediminicolota, we searched public amplicon sequencing datasets in the IMNGS database^44^ for the 16 S rRNA gene sequences of our high-quality MAGs (See Materials and Methods for details). *Ca*. Nitrosediminicolota is present with > 0.1% relative abundances in 300 globally-distributed samples (Supplementary Data 2), which are mapped in Fig. 2C. Except for two soil and 13 basaltic rock samples (from the Dorado outcrop^45^ and North Pond^46^), the vast majority of the *Ca*. Nitrosediminicolota-containing samples are marine sediments (Fig. 2D). All of the marine sites are oligotrophic sediments beneath the oligotrophic gyres of the Pacific^47^, Atlantic^17^, and Indian Oceans^48^, mid-ocean ridges^19,38,39^, hadal trenches^40,49^, and the Gulf of Mexico^50^ (Fig. 2C). The distribution of the *Ca*. Nitrosediminicolota phylum suggests it harbors microbes specialized for oligotrophic marine sediments.

### *Ca*. Nitrosediminicolota bacteria contain all key genes of nitrite oxidizers

**Fig. 3:**
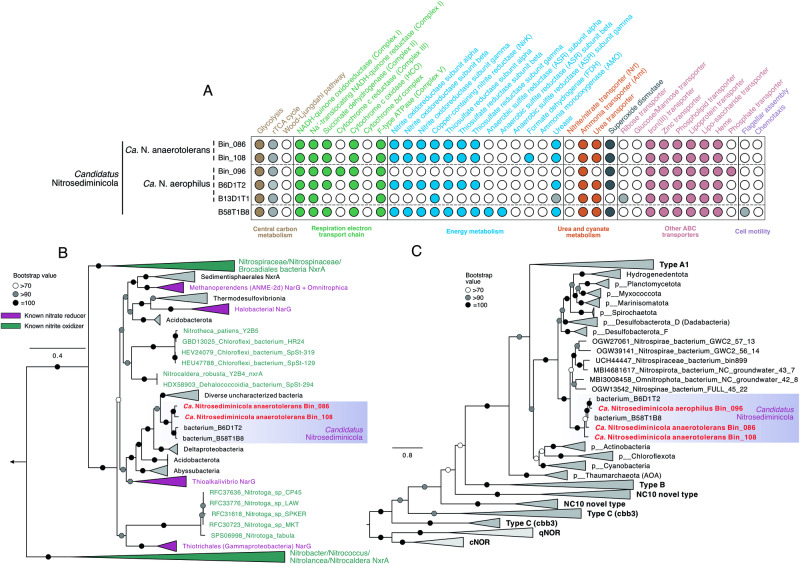
Metabolic potential of *Ca*. Nitrosediminicolota bacteria. **A** Heatmap showing the important metabolic pathways encoded by the six *Ca*. Nitrosediminicolota genomes. The filled circles indicate the presence of the full pathways, the open ones denote the absence, while the grey ones represent that the pathways are incomplete. **B** Phylogeny of NxrA/NarG of the novel NOB. The tree is rooted to two NarG sequences of NC10 bacteria. Genomes recovered in this study are shown in red. Bacteria known for having the capacity of nitrite oxidation (i.e., nitrite-oxidizing bacteria of the genera of *Nitrospira*, *Nitrospina*, *Nitrotoga*, *Nitrobacter*, and *Nitrococcus*, and anammox bacteria of the Brocadiales order) are highlighted in green. Bacteria with an observed nitrate-reducing phenotype are shown in purple. **C** Maximum-likelihood phylogenetic tree of heme copper reductase (or cytochrome *c* oxidase). The sequences of *Candidatus* Nitrosediminicolota are highlighted in a colored box, and the MAGs recovered from AMOR sediments are shown in red. For both trees, bootstrap values of > 70 (*n* = 1000) are shown with symbols listed in the legend. The scale bars show estimated sequence substitutions per residue.

*Ca*. Nitrosediminicolota members contain nitrite oxidoreductase (NXR), the key enzyme for nitrite oxidation in microorganisms. NXR is present in four *Ca*. Nitrosediminicola MAGs (Bin_086, Bin_108, B6D1T2, and B58T1B8) that span all three species in this genus (Fig. 3A). Considering the high similarities ( > 95% AAI) among the three MAGs represented by B6D1T2, it is likely that the absence of NXR in the other MAGs (Bin_096 and B13D1T1) of this species is due to their lower genome completeness (Table 1). The NXR operons in all NXR-containing *Ca*. Nitrosediminicolota genomes except B58T1B8 are present in the middle of scaffolds with lengths between 31 and 139 kbp (Fig. S4). Also, the gene arrangements around NXR in these *Ca*. Nitrosediminicolota genomes reconstructed from different geographic locations are generally consistent, suggesting that the NXRs are unlikely to be erroneously binned from other microbes. The structure of the putative NXR operons within *Ca*. Nitrosediminicolota genomes, consisting of NxrABC and a chaperone subunit annotated as NxrD (Fig. S4), is similar to those observed in Chloroflexota (*Ca*. Nitrocaldera robusta and *Ca*. Nitrotheta patiens^11^) and *Nitrotoga* NOB. Upstream of the NXR operon in the *Ca*. Nitrosediminicolota genomes are genes encoding thiosulfate reductase and arsenite oxidase (Fig. S4). Like known NXR, these two enzymes are molybdenum-containing oxidoreductases. The genes downstream of the NXRs encode cysteine desulfurase (iscS), a Fe-S cluster assembly scaffold protein (iscU), and Fe-S cluster assembly chaperones (hscAB), which are involved in the formation of Fe-S clusters^51^, a critical part of many molybdenum-containing oxidoreductases including NXR. The putative NXR may enable members of *Ca*. Nitrosediminicola to generate energy from nitrite-nitrate interconversion.

Aerobic NOBs need oxygen as their terminal electron acceptor. Five of the six *Ca*. Nitrosediminicola genomes contain a cytochrome *c* oxidase (CoxABCDE) (i.e., heme-copper oxygen (HCO) reductase) (Fig. 3A), a critical enzyme involved in oxygen respiration, while its absence in the sixth (B13D1T1) could be due to the lower genome completion level. Phylogenetic analysis of cytochrome *c* oxidase indicates that the sequences of *Ca*. Nitrosediminicola form a clade separated from other bacterial phyla and fall within the broad branch of the A1 Clade of heme-copper oxygen reductase (Fig. 3C). *Ca*. Nitrosediminicola members lack the cytochrome *bd*-type oxidases that are common in *Nitrospinaceae*^5,52^ and *Nitrospiraceae*^53^ or *cbb3*-type cytochrome *c* oxidase. The cytochrome *c* oxidase can receive electrons from NXR for aerobic respiration, and the protons released by this process can help to maintain the proton gradient that drives the ATP synthesis in Complex V. The presence of oxygen-respiring cytochrome *c* oxidase likely also enables them to complete the electron-transport chain and support the high abundances of *Ca*. Nitrosediminicola in oxic sediments.

Characterized NOBs have been suggested to acquire the NXR module in different evolutionary pathways and the horizontal transfer of NXR is likely a major driver for the spread of the capability to gain energy from nitrite oxidation during bacterial evolution^9,11,52,54,55^. In particular, the canonical marine aerobic NOBs affiliated to the genera *Nitrospira* and *Nitrospina* are suggested to obtain their NXR from anammox bacteria in the Brocadiales order within the Planctomycetota phylum^41^. Considering the distinct phylogenetic affiliations between the newly found *Ca*. Nitrosediminicola and the canonical NOB, we checked whether they acquired the nitrite oxidation capacity through the same evolutionary path. As with the NXR gene subunit organization (nxrABCD), the specific amino acid sequences of the NXR alpha subunit (NxrA) suggested that the four NXR-bearing *Ca*. Nitrosediminicola members are more similar to the recently characterized NOB of *Nitrotoga*^55–57^ and Chloroflexota (*Ca*. Nitrocaldera robusta and *Ca*. Nitrotheca patiens^11^) than *Nitrospira* and *Nitrospina* (Fig. 3B). *Ca*. Nitrosediminicolota members thus may have acquired NXR from a donor similar to Chloroflexota and *Nitrotoga* NOB rather than that of taxa within the *Nitrospiraceae*/*Nitrospinaceae*/Anammox clade.

All but one (Bin_096) of the *Ca*. Nitrosediminicola genomes encode a copper-containing nitrite reductase (NirK) (Fig. 3A), which can reduce nitrite to nitric oxide and is present in some NOBs^6,54,58^. On the maximum-likelihood phylogenetic tree of bacterial NirK *Ca*. Nitrosediminicola genomes form an independent cluster (Fig. S5). The close relatives of *Ca*. Nitrosediminicola NirK are all from ultra-small-celled archaea affiliated with *Ca*. Woesearchaeota, rather than Nitrospinota, Nitrospinota_B, Tectomicrobia, UBA8284, or Schekmanbacteria, indicating that NirK in *Ca*. Nitrosediminicola may have a different origin than the majority of the *Ca*. Nitrosediminicola genes.

**Fig. 4:**
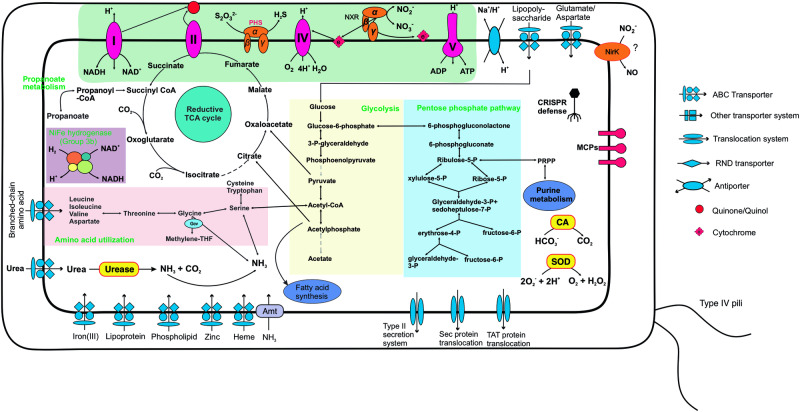
Potential key metabolic interactions in *Ca*. Nitrosediminicolota bacteria. The common metabolic pathways in the *Ca*. Nitrosediminicolota bacteria include aerobic respiration, nitrite oxidation (NXR), oxygen respiration (Complex IV), urea assimilation and hydrolysis, reductive TCA cycle, nitrite reduction (NirK), glycolysis, superoxide dismutase (SOD), pentose phosphate pathway, and ABC transport for iron, zinc, heme, lipoprotein, and phospholipid.

For the full respiratory electron-transport chain, *Ca*. Nitrosediminicola genomes have Complex I, Complex II, Alternative Complex III, Complex IV (described above), and Complex V (F-type ATPase) (Fig. 3A), like other previously characterized aerobic NOB. This complete oxygen respiratory electron-transport chain likely enables them to oxidize nitrite under oxic conditions. Regarding the central carbon metabolism, similar to the two newly-cultured NOBs affiliated with *Nitrospinaceae* from coastal sediments^5^, *Ca*. Nitrosediminicola species encode most key genes of the reductive tricarboxylic acid (rTCA) cycle (Figs. 3 and 4), including the hallmark enzymes 2-oxoglutarate:ferredoxin oxidoreductase and pyruvate:ferredoxin oxidoreductase. Similar to nitrite-oxidizing Chloroflexota^11^, the ATP-citrate lyase is absent, whose function could be replaced by the reversibility of the encoded citrate synthase^59,60^. The rTCA cycle may enable *Ca*. Nitrosediminicola bacteria to fix CO_2_ as proposed previously for *Nitrospira* and *Nitrospina*^52,54,61^. The electrons for carbon fixation may be derived from nitrite oxidation^52^. The *Ca*. Nitrosediminicola genomes also encode the gluconeogenesis and the pentose phosphate pathways (Fig. 4), which may be employed for the synthesis of precursor metabolites in these NOBs, as previously proposed for *Nitrospira moscoviensis*^61^. Another feature of *Ca*. Nitrosediminicola is that five of its six member genomes contain a urease operon (Fig. 3A and Fig. S6) (See Supplementary Note 1), which may enable them to access this pool for substrates and engage in reciprocal feeding with co-occurring ammonia-oxidizing archaea^16,25^ to increase their metabolic fitness in marine sediments. The ABC transporters of iron(III), zinc, phospholipids lipoprotein, and heme are conserved in *Ca*. Nitrosediminicolota genomes (Fig. 3A). Like many sediment bacteria, they lack genes for flagellar assembly and chemotaxis.

*Ca*. Nitrosediminicolota members more likely employ the putative NXR to perform nitrite oxidation rather than heterotrophic denitrification, due to the following reasons. First, they mainly inhabit oligotrophic deep-sea sediments (Fig. 2C), which typically contain limited organic matter and deep oxygen penetration, and what organic matter that is available is largely inaccessible to heterotrophs through protection by mineral adsorption^62^. Second, their genomes contain no genes involved in the transport of oligosaccharides, monosaccharides, or amino acids, and only one genome (Bin_108) contains formate dehydrogenase, indicating that their capacity for organic matter respiration is minimal. Because the absence of sulfide and methane in the investigated AMOR sediments^63^, they are also unlikely to be capable of autotrophic denitrification. While all members of *Ca*. Nitrosediminicolota reported here appear to retain nitrite oxidation capacity, conclusively including all *Ca*. Nitrosediminicolota among NOB requires cultures to prove biogeochemical function, especially under anoxic conditions.

### *Ca*. Nitrosediminicolota resolves the nitrifier abundance discrepancy

Given the likely nitrite oxidation capacity of the newly defined *Ca*. Nitrosediminicolota, we re-calculated the abundances of combined NOB (defined as the sum of *Nitrospiraceae*, *Nitrospinaceae*, and *Ca*. Nitrosediminicolota) in the sediment cores by including these as part of the NOB community (Supplementary Data 1). In the five Atacama Trench sediment cores, the abundances of AOA vs. NOB in the oxic zones did not significantly change and still fell into the theoretical range of 2.6–15.8, with a mean AOA:NOB abundance ratio of 7.3 (Fig. 1B). For the 11 cores from the Arctic and Atlantic Ridges, however, the updated abundance ratios of AOA and NOB fell closer to the theoretical range (Fig. 1D), with a mean AOA:NOB abundance ratio of 9.6. The median of AOA:NOB ratio of these 82 oxic samples decreased dramatically from 43.3 to 5.6, with a 99% confidence interval of 3.9–8.4 (Fig. 1E). These results align with the theoretical prediction based on the observed growth features of marine AOA and NOB. Admittedly, microbes in deep-sea sediments face extreme energy limitation and are generally sustained by basal power requirements^64^. Therefore, higher bulk reaction rates are typically accompanied by higher microbial abundances across samples of different depths/ages, because the underlying microbes have similar power requirements [e.g., AOA^28^ and sulfate-reducing bacteria^65^]. Although other factors such as transcription may also influence the relative abundance of AOA and NOB, our quantitative data indicate that counting these novel bacteria as NOBs helps resolve the apparent abundance mismatch between AOA and NOB in marine sediments.

**Fig. 5:**
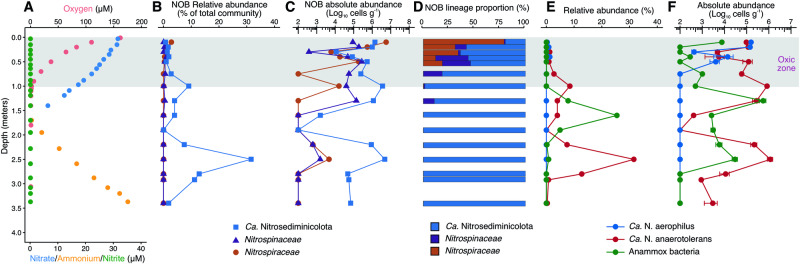
Geochemical context, relative abundances, and community compositions of NOB lineages in AMOR core GS14-GC08. **A** Geochemical context delineated by the measured profiles of oxygen, nitrate, nitrite, and ammonium, previously reported in ref. 28. The oxic zone is marked with a grey box. **B** The relative abundances of *Ca*. Nitrosediminicolota and the canonical marine NOB families *Nitrospiraceae* and *Nitrospinaceae*, as assessed by amplicon sequencing. **C** The absolute abundances of the three NOB lineages calculated as the product of the relative abundances of the three lineages and the total cell numbers. **D** The putative NOB community composition in each investigated depth. **E**, **F** The relative (**E**) and absolute (**F**) abundances of two *Ca*. Nitrosediminicola species (*Ca*. N. aerophilus and *Ca*. N. anaerotolerans) and anammox bacteria throughout the core. The same data for other three AMOR cores are shown in Fig. S7.

To check whether *Ca*. Nitrosediminicolota is the dominant nitrite oxidizer in both oxic and anoxic marine sediments, we compared the abundances of *Ca*. Nitrosediminicolota to those of canonical NOB affiliated with the families *Nitrospinaceae* and *Nitrospiraceae* in AMOR sediment cores. Although these two NOB families, especially *Nitrospinaceae*, are also abundant in oxygen-deficient waters^35^, in AMOR sediments they are generally confined within the oxic zones with <4% relative abundances among the total prokaryotic communities (Figs. 5A, 5B, and Fig. S7B). In contrast, *Ca*. Nitrosediminicolota is present in most of the investigated depths and is particularly abundant in the deep anoxic layers. Restricting analysis to the oxic zone (Fig. 5A) where the organisms co-occur, *Ca*. Nitrosediminicolota dominates over *Nitrospinaceae* and *Nitrospiraceae* in all but a few depths (Fig. 5D and Fig. S7D). The depth-averaged relative abundance of *Ca*. Nitrosediminicolota in the putative NOB communities in oxic sediments of the 11 cores is 50–80%, while *Nitrospiraceae* and *Nitrospinaceae* each only account for 8–25% (Fig. 1F). Thus, *Ca*. Nitrosediminicolota is roughly 2–4 times more abundant than the canonical NOBs in oxic marine sediments. The dominance of *Ca*. Nitrosediminicolota is also evident in the calculated absolute abundance profiles in the four AMOR cores (Fig. 5C and Fig. S7C), who exhibit 2–4 orders of magnitude higher absolute abundances than *Nitrospinaceae* and *Nitrospiraceae* in the basal part of the oxic zones. Being far more abundant than canonical NOBs, *Ca*. Nitrosediminicolota can potentially contribute significantly to nitrite oxidation in global oligotrophic marine oxic sediments.

### Redox niches distinguish *Ca*. Nitrosediminicola species

To reveal which *Ca*. Nitrosediminicolota species are present and can thrive in the anoxic sediment layers of the AMOR cores, we interrogated the distribution of individual species represented by the *Ca*. Nitrosediminicola MAGs in the four AMOR cores previously reported^38^. Based on the comparison of 16 S rRNA gene sequences between *Ca*. Nitrosediminicola MAGs and the amplicon sequencing OTUs (See Materials and Methods), *Ca*. Nitrosediminicola Bin_086 and *Ca*. Nitrosediminicola Bin_096 reported here correspond to OTU_4 and OTU_235 reported in Zhao et al.^38^, respectively. It is worth noting that these two OTUs could be classified as members of the Schekmanbacteria phylum (SILVA 138.1) or the Nitrospinota phylum (MD2896-B214 class, SILVA 128), depending on the reference databases used in the classification. The matches between the MAGs reported here and the previously reported OTUs set the basis for tracking the vertical distribution of the two *Ca*. Nitrosediminicola species in the four AMOR cores.

We observed distinct redox niche preferences of the two *Ca*. Nitrosediminicola species derived from the AMOR cores. While *Ca*. Nitrosediminicolota Bin_096 (OTU_235) is exclusively detected in the oxic zones of the four AMOR cores, *Ca*. Nitrosediminicolota Bin_086 (OTU_4) was detected in all investigated sediment layers and is particularly abundant in anoxic layers (Fig. 5E and Fig. S7E). Therefore, *Ca*. Nitrosediminicolota Bin_096 appears to be an oxic niche specialist, while *Ca*. Nitrosediminicolota Bin_086 may be an anoxia-tolerant generalist. The high relative abundance of *Ca*. Nitrosediminicolota in the deep anoxic layers of the AMOR cores (Fig. 5B and Fig. S7B) is due to the prevalence of *Ca*. Nitrosediminicola Bin_086 in the anoxic sediments. The maximum relative abundance of *Ca*. Nitrosediminicola Bin_086 (31% of the total community) was detected at 250 cm below the seafloor of GS14-GC08 (Fig. 5B). Whether *Ca*. Nitrosediminicola Bin_086 can perform nitrite oxidation in the deep anoxic sediment layers requires future study. Such a redox niche preference difference between different lineages of the same functional guild is not novel, and has been previously observed for anammox bacterial families in AMOR sediments^19^. To reflect their preferred redox niches, we propose to name Bin_096 *Ca*. Nitrosediminicola aerophilus (prefer aerobic conditions), and Bin_086 *Ca*. Nitrosediminicola anaerotolerans (tolerant to anaerobic conditions).

The abundant *Ca*. N. anaerotolerans may compete with anammox in anoxic layers. In GS14-GC08, the relative and absolute abundances of *Ca*. N. anaerotolerans exhibited two peaks in the anoxic sediments (Fig. 5E, F), which are located above and below the nitrate-depletion zone. Interestingly, the peak (of both the relative and absolute abundances) of anammox bacteria was observed between the two *Ca*. N. anaerotolerans abundance maxima, which appears to indicate potential competition between these two nitrite-consuming groups. Although the two abundance peaks of *Ca*. N. anaerotolerans are not resolved in the other AMOR cores (GS14-GC09, GS16-GC04, and GS16-GC05) due to their short core length or low depth resolution, they generally show that the abundance of *Ca*. N. anaerotolerans decreases along with the increase of anammox bacteria abundance in anoxic sediments (Fig. S7) (Supplementary Data 3). Although *Ca*. N. anaerotolerans is likely involved nitrite oxidation in the upper oxic sediments, it remains unclear whether they can maintain the same capacity or need nitrite for other purposes in layers without detectable oxygen. Therefore, whether *Ca*. N. anaerotolerans competes for nitrite with anammox bacteria is still unclear. Nevertheless, our observation suggests that anammox bacteria retain better fitness over *Ca*. N. anaerotolerans in anoxic sediments where nitrate and nitrite supply may be limiting.

To identify what mechanisms may drive the distinct redox niche preferences between the two prevailing Nitrosediminicolota species, we performed a comparative genomic analysis based on four MAGs (*Ca*. N. anaerotolerans represented by Bin_086 and Bin_108, and *Ca*. N. aerophilus represented by Bin_096 and B6D1T2). A total of 5928 genes of the four MAGs form 1355 gene clusters. There are 34 (2.5% of the total) gene clusters uniquely present in *Ca*. N. anaerotolerans and 37 (2.7%) in *Ca*. N. aerophilus, while 989 (73.0%) gene clusters are shared among the four Nitrosediminicolota genomes (Supplementary Data 4). Thiosulfate reductase is among the enzymes encoded by the gene clusters uniquely present in *Ca*. N. anaerotolerans. Thiosulfate is a common sulfur cycle intermediate at low concentrations in marine sediments and is mainly produced by the oxidation of hydrogen sulfide derived from sulfate reduction^66,67^. The presence of thiosulfate reductase in *Ca*. N. anaerotolerans may equip it with the capacity to use thiosulfate as an electron acceptor in anoxic sediments. In addition, unlike *Ca*. N. aerophilus, *Ca*. N. anaerotolerans also contains genes involved in cobalamin (Vitamin B12) synthesis. Unique gene clusters in the *Ca*. N. aerophilus genomes encode ABC-type proline/glycine betaine transporters for bacterial osmoregulation and Mu-like prophage proteins involved in bacterial antiviral defense^68^ (Supplementary Data 4). Other gene clusters found to be uniquely present in only one Nitrosediminicolota species have no known roles in energy metabolism.

## Conclusion

Our compilation of AOA and canonical NOB abundances in global oxic marine sediments argues that there were overlooked yet abundant NOB. Through genome reconstruction and phylogenetic analyses, we discovered a bacterial phylum, *Ca*. Nitrosediminicolota, which currently contains six genomes that can be resolved to three species in the same genus *Ca*. Nitrosediminicola. Metabolic potential analyses of *Ca*. Nitrosediminicolota genomes indicated that they contain the genetic machinery for nitrite oxidation as well as other versatile metabolisms such as urea utilization. *Ca*. Nitrosediminicolota is widespread in oligotrophic marine sediments. They are more abundant in the oxic zones of AMOR sediments by a factor of 2–4 compared to the canonical NOBs affiliated with *Nitrospiraceae* and *Nitrospinaceae*. Counting them as NOB resolves the abundance mismatch between AOA and NOB in broad oxic marine sediments and permits closing the nitrogen cycle in oxic marine sediments without invoking denitrification. Although being affiliated with the same genus, the two dominant *Ca*. Nitrosediminicola species in the AMOR sediments manifest distinct redox niche preferences: *Ca*. N. aerophilus is only present in the oxic zone whereas *Ca*. N. anaerotolerans exist both in the oxic and anoxic zones. Its capacity for thiosulfate reduction may allow *Ca*. N. anaerotolerans to thrive under anaerobic conditions but cultivation efforts are needed to confirm these genome-based metabolic inferences. Considering their global occurrence and high abundance in sediments not only on the Arctic ridge but also beneath open ocean gyres and in hadal trenches, *Ca*. Nitrosediminicolota may play a critical role in sediment nitrogen cycling across the entire oligotrophic marine expanse.

### Etymology description

*Candidatus* Nitrosediminicolota (Ni.tro.se.di.mi.ni.co.lo’ta. N.L. masc. n. Nitrosediminicola, a bacterial genus; -ota, ending to denote a phylum; N.L. neut. pl. n. Nitrosediminicolota, the Nitrosediminicola phylum).

*Candidatus* Nitrosediminicola (Ni.tro.se.di.mi.ni’co.la. Gr. neut. n. nitron, mineral alkali; L. neut. n. sedimen, sediment; L. masc./fem. n. suff. -cola, inhabitant, dweller; N.L. masc. n. Nitrosediminicola, a nitrate forming sediment-dweller).

*Candidatus* Nitrosediminicola aerophilus (aero, oxygen; suff. -philus, lovers; aerophilus, an oxygen lover, highlighting the preference of this microbe to the oxic zone of marine sediments).

Phylogenetically affiliated with the genus *Ca*. Nitrosediminicola, phylum *Ca*. Nitrosediminicolota. This species currently contains three genomes from marine sediments (two from the Mariana Trench and one from the Arctic Mid-Ocean Ridge). The arctic genome consists of 73 scaffolds of 1,837,265 bp. The DNA G + C content is 60.6%. It is preferably present in the oxic sediment layers. It contains metabolic functions of aerobic nitrite oxidation and urea hydrolysis.

*Candidatus* Nitrosediminicola anaerotolerans (anaero, lack of oxygen; suff. -tolerans, being tolerant to something; anaerotolerans, being tolerant to anaerobic conditions, highlighting the tolerance of this microbe to the anoxic zone of sediment columns)

Phylogenetically affiliated with the genus *Ca*. Nitrosediminicola, phylum *Ca*. Nitrosediminicolota. This species contains two strains recovered from two Arctic sediment cores. Their genomes consist of 48–61 scaffolds, with total genome sizes of 2.2–2.5 Mbp. The DNA G + C content is 62.3%. The genomes are present in both the oxic and anoxic sediment layers. It contains metabolic functions of nitrite-nitrate conversion, urea hydrolysis, and thiosulfate reduction.

## Methods

### Sampling collection and characterization

This study uses samples and data of sediment cores from the Arctic, Atlantic, and Pacific Oceans. The procedures of sample collection, processing, and data generation were thoroughly described in refs. ^16,17,38,39^. Briefly, sediment cores were retrieved by gravity coring from the seabed of various sites on the ridge flanks of the Arctic Mid-Ocean Ridge beneath the Norwegian-Greenland Sea^38,39^ or by piston coring from the North Atlantic Ocean^16,17^. Upon core retrieval, the thickness of the oxic zone of each core was determined by measuring the in-situ oxygen concentrations using a needle-type fiber-optic oxygen microsensor (PreSens), except for GS13-CC2 in which the oxygen penetration depth was not measured but inferred as the depth marking the appearance of dissolved Mn in the porewater^41^. For the Arctic cores where the distribution patterns of *Ca*. Nitrosediminicola species were investigated in this study, the subsampling of microbiology samples (using sterile 10 mL cutoff syringes) and porewater extraction were performed immediately on the sampling half using Rhizons samplers after the split. A QuAAtro 114 continuous flow analyzer (SEAL Analytical Ltd) was used to colorimetrically measure nitrate, nitrite, and ammonium concentrations in the porewater.

### Exploring AOA and NOB abundances in marine oxic sediments

Similar to Zhao et al.^28^ where AOA’s distribution was explored, we investigated the distribution of NOB in oxic marine sediments based on the existing 16 S rRNA gene amplicon sequencing data for 11 sediment cores with thick oxic zones. In addition to the cores considered in Zhao et al.^28^, we also included four additional AMOR cores (GS13-CC2, GS14-GC02, GS14-GC04, and GS15-GC01 reported in refs. ^39,41^) and two piston cores from the North Atlantic Gyre^17^. The amplicon sequencing data of the total eight AMOR cores and the North Pond core were generated using the same procedure. Briefly, the total DNA in the sediment samples was extracted using the PowerLyze DNA extraction kits (MOBIO Laboratories, Inc.). Amplicon of the 16 S rRNA gene was prepared using the two-round PCR amplification strategy with the “universal” primers of Uni519F/806r, as described in Zhao et al.^38^. The amplicon libraries were sequenced on an Ion Torrent Personal Genome Machine. The raw sequencing reads were quality filtered and trimmed to 220 bp using the USEARCH v11.0.667 pipeline^69^. The taxonomic classification of OTUs was performed using the lowest common ancestor algorithm implemented in the Python version of CREST4 (the latest version of CREST^70^) against the SILVA 138.1 Release^71^. The total cell numbers were taken as the sum of the archaeal and bacterial 16 S rRNA genes as determined by qPCR. For the remaining two cores from the North Atlantic Gyre^17^, we downloaded the amplicon sequencing data from the NCBI database and employed the same data analysis pipeline to run the reads trimming, OTU clustering and classification.

We initially considered the abundance of canonical NOB affiliated with the families *Nitrospiraceae* and *Nitrospinaceae*. For both AOA and canonical NOB, the absolute abundance of a functional group was calculated as the product of the total cell numbers (the sum of archaeal and bacterial 16 S rRNA gene abundances) and its relative abundances in the total communities (as assessed by 16 S rRNA gene amplicon sequencing), as previously employed for investigation of anammox bacteria^38,72^. We then also considered members of *Ca*. Nitrosediminicolota as some overlooked NOB in marine sediments and calculated the total NOB abundance by taking NOB abundance as the sum of *Nitrospiraceae*, *Nitrospinaceae*, and *Ca*. Nitrosediminicolota.

We also investigated the community structure of NOB based on the 16 S rRNA gene amplicon sequencing data. Through the phylogenetic analysis of 16 S rRNA gene sequences (see the description below), we confirmed that 10 OTUs were affiliated to the *Nitrospiraceae* family, 14 OTUs *Nitrospinaceae*, and 8 OTUs originally classified as members of the Schekmanbacteria phylum should correspond to the *Ca*. Nitrosediminicolota phylum. Note that among the 8 putative *Ca*. Nitrosediminicolota OTUs, only three were verified to be members of *Ca*. Nitrosediminicolota by the phylogenetic analysis of the 16 S rRNA gene (Fig. 2B) and therefore were included in the abundance calculations of *Ca*. Nitrosediminicolota, while the remaining five minor OTUs were affiliated with other bacterial phyla. For each of these three groups, the relative abundance was taken as the sum of the relative abundances of the corresponding OTUs. To quantitatively evaluate the dominance of these three putative NOB lineages based on the observed depth profiles from arbitrarily selected sediment depths, we calculated the depth-averaged relative abundance for each of the three lineages using trapezoidal integration, as implemented in the R package *pracma* (https://github.com/cran/pracma).

### Genome binning and refinement

For metagenome-assembled genome recovery, we focused on the metagenome sequencing data of core GC08 and NP-1383E, which were generated and reported by ref. ^38^. The procedures for DNA extraction, library preparation, metagenome sequencing, raw data quality control, assembly, and genome binning were described therein. Briefly, DNA was extracted from ~7 g of sediment from each selected depth. Metagenomic libraries were sequenced (2 × 150 bp paired-end reads) by an Illumina HiSeq 2500 sequencer. The quality of the raw sequencing data was first checked using FastQc v0.11.9^73^, with the adapters removed and reads trimmed using Trimmomatic v0.39^74^ based on the quality scores. The quality-controlled paired-end reads were de novo assembled into contigs using MEGAHIT v1.1.2^75^ with the *k*-mer length varying from 27 to 117. Contigs larger than 1000 bp were automatically grouped into genome bins using MaxBin2 v2.2.5^76^ and MetaBAT v2.15.3^77^ with the default settings, and the best representatives were selected using DAS_Tool v1.16^78^. The quality of the obtained bins was assessed using CheckM2 v1.0.2^79^.

In this study, three putative NXR-containing MAGs (Bin_086, Bin_096, and Bin_108) were found to be affiliated with unknown bacterial phyla and were thus subject to further analyses. To ensure the binning correctness and also improve the MAG quality, quality-trimmed reads of the sample showing the highest genome coverage were mapped onto the contigs using BBmap^80^, and the successfully aligned reads were re-assembled using SPAdes v3.12.0^81^ with the *k*-mers of 21, 33, 55, and 77. After the removal of contigs shorter than 1000 bp, the resulting scaffolds were visualized and manually re-binned using gbtools v2.6.0^82^ based on the GC content, taxonomic assignments, and differential coverages of contigs across multiple samples, with the input data generated using the following steps. Coverages of contigs in each sample were determined by mapping trimmed reads onto the contigs using BBMap v.37.61^80^. The taxonomic classification of contigs was assigned by BLASTn^83^ according to the taxonomy of the single-copy marker genes in contigs. SSU rRNA sequences in contigs were identified using Barrnap^84^ and classified using VSEARCH^85^. The mapping, re-assembly, and re-binning process was repeated 5–7 times until the quality of the genomes could not be improved further. The refined MAGs were classified using GTDB-tk v2.3.0^86^ with the default setting. The MAG quality was checked again using CheckM2 v1.0.2^79^.

### Genome annotation

Genomes discussed in this study were annotated together with their close relative MAGs [i.e., three MAGs recovered from Mariana Trench sediments^42^] and also representative MAGs in the phyla Nitrospinota and Nitrospinota_B in the GTDB 08-RS214 Release (https://gtdb.ecogenomic.org/). Genes in these genomes were predicted using Prodigal^87^. Genome annotation was conducted using Prokka v1.13^88^, eggNOG^89^, and BlastKoala^90^ using the KEGG database. The functional assignments of genes of interest were also confirmed using BLASTp^84^ against the NCBI RefSeq database. The metabolic pathways were reconstructed using KEGG Mapper^91^. The gene organizations around NXR in *Ca*. Nitrosediminicolota and also other selected NOBs in the Nitrospirota, Nitrospinota, and Chloroflexota phyla were visualized using GeneSpy v1.2^92^, with the gff files from the Prokka annotation as the input.

### Linking MAGs with amplicon sequencing OTUs

To track the vertical distribution pattern of the two *Ca*. Nitrosediminicola species in the four AMOR cores, we searched the corresponding OTUs of the two genomes by comparing their 16 S rRNA gene sequences (i.e., the query sequences) with the amplicon sequencing OTUs (the subject sequences) with BLASTp^93^. Because Bin_096 reconstructed from AMOR sediments lacked a 16 S rRNA gene sequence, we used that of B6D1T2 (another strain highly similar to Bin_096) to run the comparison. *Ca*. Nitrosediminicola Bin_086 has a full-length (1,565 bp) 16 S rRNA gene sequence, which is a 100% match with OTU_4. *Ca*. Nitrosediminicola Bin_096 corresponds to OTU_235, given the 99.6% match of the 16 S rRNA gene between them.

### Comparative genomic analysis

We performed a comparative analysis on the three representative genomes of the two *Ca*. Nitrosediminicola species using Anvi’o v7.1^94^ according to the pangenome analysis workflow. All genomes were first annotated using Prokka v.1.14^88^ and BLASTp using the Clusters of Orthologous Groups of Proteins (COG)^95^ as the reference database. The comparative genomic analysis uses BLAST to quantify the similarity between each pair of genes, and the Markov Cluster algorithm (MCL)^96^ (with an inflation parameter of 2) to resolve clusters of homologous genes. The shared and unique genes in the two genomes were identified via the functional enrichment analysis^97^. Average amino acid identities between genomes were calculated using EzAAI v.1.2.2^98^ with the default setting.

### Phylogenetic analyses

To pinpoint the phylogenetic placement of the newly recovered MAGs and their relative genomes, we performed phylogenetic analyses for them together with high-quality genomes that were included in the GTDB Release 08-RS214. The 120 single-copy genes were identified, aligned, and concatenated using GTDB-tk v2.3.0^86^ with the “classify_wf” command. The maximum-likelihood phylogenetic tree was inferred based on this alignment using IQ-TREE v1.5.5^99^ with LG + F + R7 the best-fit model selected by ModelFinder^100^, and 1000 ultrafast bootstrap iterations using UFBoot2^101^. To provide support to this phylogenomic analysis, we also performed the phylogenomic analysis based on the 14 syntenic ribosomal proteins (rpL2, 3, 4, 5, 6, 14, 16, 18, 22, and rpS3, 8, 10, 17, 19). These selected proteins were identified in Anvi’o v7.1^94^ using Hidden Markov Model (HMM) profiles and aligned individually using MUSCLE^102^. Alignment gaps were removed using trimAl^103^ in “automated” mode and the individual alignments of ribosomal proteins were concatenated. The maximum likelihood phylogenetic tree was reconstructed using IQ-TREE v1.5.5^99^ with LG + R7 as the best-fit model.

A maximum-likelihood phylogenetic tree based on 16 S rRNA genes was also constructed for the above-mentioned genomes to confirm the phylogenetic placement of the *Ca*. Nitrosediminicolota phylum. To expand this phylum on the tree beyond the available genomes, the putative *Ca*. Nitrosediminicolota OTUs from the amplicon sequencing and their close relatives identified via BLASTn^93^ in the NCBI database were also included. Sequences were aligned using MAFFT-LINSi^104^ and the maximum-likelihood phylogenetic tree was inferred as above, with 1000 ultrafast bootstraps.

For the phylogenies of NxrA (nitrite oxidoreductase alpha subunit), the *Ca*. Nitrosediminicola sequences were used as the queries in BLASTp^93^ searches in the NCBI database ( > 50% similarity and *E*-value of 10^−^^6^) to identify their close relatives. These sequences were aligned using MAFF-LINSi^104^ with reference sequences from Koch et al.^105^ and complemented with known nitrite-oxidizing bacteria. For the phylogeny of UreC (urease alpha subunit), the sequences of *Ca*. Nitrosediminicola genomes were used as the queries in the BLASTp^93^ search in the NCBI database (only hits of >50% similarity were retained), to identify their close relatives. These sequences were combined with sequences from Zhao et al.^41^ and were aligned using MAFF-LINSi^104^. The same procedure was also used to prepare sequences for the phylogenetic analyses of NirK (copper-containing nitrite reductase) and heme copper oxygen reductase. Phylogenetic trees for all proteins were generated as above.

### Global occurrence of *Ca*. Nitrosediminicolota

The global occurrence of *Ca*. Nitrosediminicolota in natural environments was assessed using IMNGS^44^ against all public SRA datasets in the NCBI database with the 16 S rRNA gene sequences of high-quality *Ca*. Nitrosediminicolota genomes as the query. Reads were counted as matching reads if they (i) were longer than 200 bp and (ii) showed >95% nucleotide sequence identity to the query. Samples with less than 10 matching reads were discarded. Only natural environments with more than 0.1% relative abundances were retained for spatial mapping. The sample coordinates were mapped onto a global map using the R packages *rgdal* and *rgeos*. The basal global map was created in R using free vector and raster map data from Nature Earth (https://www.naturalearthdata.com/).

### Statistics and reproducibility

Statistical analyses were performed in R v4.2.2^106^. The linear correlations between AOA and NOB abundances were calculated using the “*lm()*” function of R. The abundance data used in this study were derived from single measurements of individual sediment samples without replicates from a total of 17 sediment cores (110 individual samples).

### Reporting summary

Further information on research design is available in the Nature Portfolio Reporting Summary linked to this article.

### Supplementary information

Supplementary Information

Description of Additional Supplementary Files

Supplementary Data 1

Supplementary Data 2

Supplementary Data 3

Supplementary Data 4

Reporting Summary

## Data Availability

All sequencing data used in this study are available in the NCBI Short Reads Archive under the project number PRJNA529480. The three *Ca*. Nitrosediminicolota genomes recovered in this study are available under the accession number JAWJBM000000000 (*Ca*. N. anaerotolerans Bin_086), JAWJBN000000000 (*Ca*. N. anaerotolerans Bin_108), and JAWJBO000000000 (*Ca*. N. aerophilus Bin_096). Microbial abundance and relevant geochemical data are available with this paper. All other data are available from the corresponding authors on reasonable request.
