# Supplementary material for: An abundant bacterial phylum with nitrite-oxidizing potential in oligotrophic marine sediments
Source: Commun Biol. 2024 Apr 11;7:449. doi: 10.1038/s42003-024-06136-2 (PMC11009272; doi:10.1038/s42003-024-06136-2)
Supplement: Supplementary file 1 — Supplementary Information [file 42003_2024_6136_MOESM1_ESM.docx]

Supplementary Information for

**An abundant bacterial phylum with nitrite-oxidizing potential**

**in oligotrophic marine sediments**

Rui Zhao^1*^, Steffen L. Jørgensen^2^, Andrew R. Babbin^1*^

^1^ Department of Earth, Atmospheric and Planetary Sciences, Massachusetts Institute of Technology, Cambridge, MA, USA

^2^ Centre for Deep-Sea Research, Department of Earth Science, University of Bergen, Bergen, Norway

*Correspondence to RZ (rzh041@mit.edu) or ARB (babbin@mit.edu)

**Table of Contents**

Supplementary Note 1

Supplementary Figures S1–S7

Supplementary References

**Supplementary Note 1**

**The capacity for urea hydrolysis in *Ca.* Nitrosediminocolota**

*Ca.* Nitrosediminocolota members can use urea as an alternative energy source. Similar to the NXR operon, the full urease operon (ureABCDFG) is present in the four *Ca.* Nitrosediminocolota genomes of higher completion (Fig. 3) and a partial urea operon in B13D1T1, while its absence in the remaining MAG (Bin_096) may be attributed to the low genomic completeness. It includes a nickel-dependent urease (UreABC) as well as accessory proteins (UreDFG) for the maturation of the holoenzyme. Additionally, all these urease-positive genomes possess a complete gene set for an ATP-dependent ABC-type urea transporter (UrtABCDE) encoded upstream of the urease structural genes. This type of transporter is characterized by its high affinity for urea, indicating an adaptation to low urea concentrations in the environment, such as marine sediments where tens of nanomolar concentrations of urea have been detected^1, 2^. In oxygenated environments where ammonium is maintained at low concentrations due to aerobic ammonia oxidation activity, urea can reach similar 10s to 100s of nanomolar levels as to ammonium^2^. In AMOR sediments, *Ca.* Nitrosediminocolota is the third phylum harboring microbes capable of urea utilization, after anammox bacteria affiliated to Planctomycetota^3, 4^ and AOA affiliated to Thaumarchaeota^5^.

*Ca.* Nitrosediminocolota bacteria may not oxidize ammonium directly because none of the five *Ca.* Nitrosediminocolota genomes has an ammonia monooxygenase. However, by providing a source of ammonium, the capacity of urea lysis of *Ca.* Nitrosediminocolota may allow reciprocal feeding with ammonia oxidizers^6^. In this substantial ecological advantage for NOBs^6^, the ammonium released from urea degradation can serve as the substrate of the ammonia oxidizers, which in turn can provide nitrite to *Ca.* Nitrosediminocolota. This ecological advantage may be important to *Ca.* Nitrosediminocolota to grow in ammonium-limited habitats such as the oxic zone of marine sediments. Urease is not universally present in NOB, and it is notably absent in the recently reported *Nitrospinaceae* and *Nitrospiraceae* NOB cultured from coastal sediments^7, 8^. The patchy distribution of urease among NOB suggests further niche differentiation of these organisms based on the capacity to use organic nitrogen compounds (e.g., urea) as sources of reduced nitrogen for assimilation.

Despite their close phylogenetic relationship, *Ca.* Nitrosediminocolota may acquire the urease via a route different from that of Nitrospinota. Phylogenetic analysis of UreC (urease alpha submit) indicates that *Ca.* Nitrosediminocolota genomes form a clade distinct from other bacterial phylum, and particularly other nitrogen cycling guilds (e.g., AOA, AOB, and NOB from the Nitrospirota and Nitrospinota phyla) (Fig. S5), suggesting that *Ca.* Nitrosediminocolota acquired urease differently from other nitrogen cycling groups. UreC sequences of *Ca.* Nitrosediminocola members show similarities to those of some Firmicutes (Fig. 3), indicating potential horizontal gene transfer (HGT) events between these two bacterial phyla given that these two phyla are not in close proximity on the tree of bacteria. It is likely that, like NXR, urease was horizontally disseminated between bacteria on multiple occasions.

**Similarity to other characterized NOBs**

Aerobic microbes display a variety of mechanisms, such as superoxide dismutase and catalase, to resist the oxidative stress caused by reactive oxygen species prevalent in oxic environments. While such canonical systems seem to be absent in some *Nitrospinaceae* genomes [e.g., refs^9, 10^], five of the six *Ca.* Nitrosediminocola genomes have superoxide dismutase (Fig. 3A), which may serve as a reactive oxygen species protection mechanism and help them to maintain anoxic niches in the oxic zone. Whether the presence of superoxide dismutase in *Ca.* Nitrosediminocola contribute to their ecological success in oxic AMOR sediments remains for further studies.

*Ca.* Nitrosediminocola members appear to lack the capacity for using formate, with formate dehydrogenase is only present in one of the six *Ca.* Nitrosediminocola MAGs. Formate dehydrogenase is present in most functionally characterized NOBs, and has been experimentally confirmed in *Nitrospira moscoviensis*^6, 11^, *Nitrospira marina*^12^*,* and *Nitrotoga fabula*^13^, in which formate is used as an energy source and electron donor with nitrate as the terminal electron acceptor^6, 11, 12^. All six *Ca.* Nitrosediminocola genomes also lack the genes for flagellar synthesis and chemotaxis (Fig. 3), indicating that they may not be able to migrate within the sediment pore space, and thus are confined to the geochemical conditions of the depth at which they reside.

**Supplementary Figures**

**
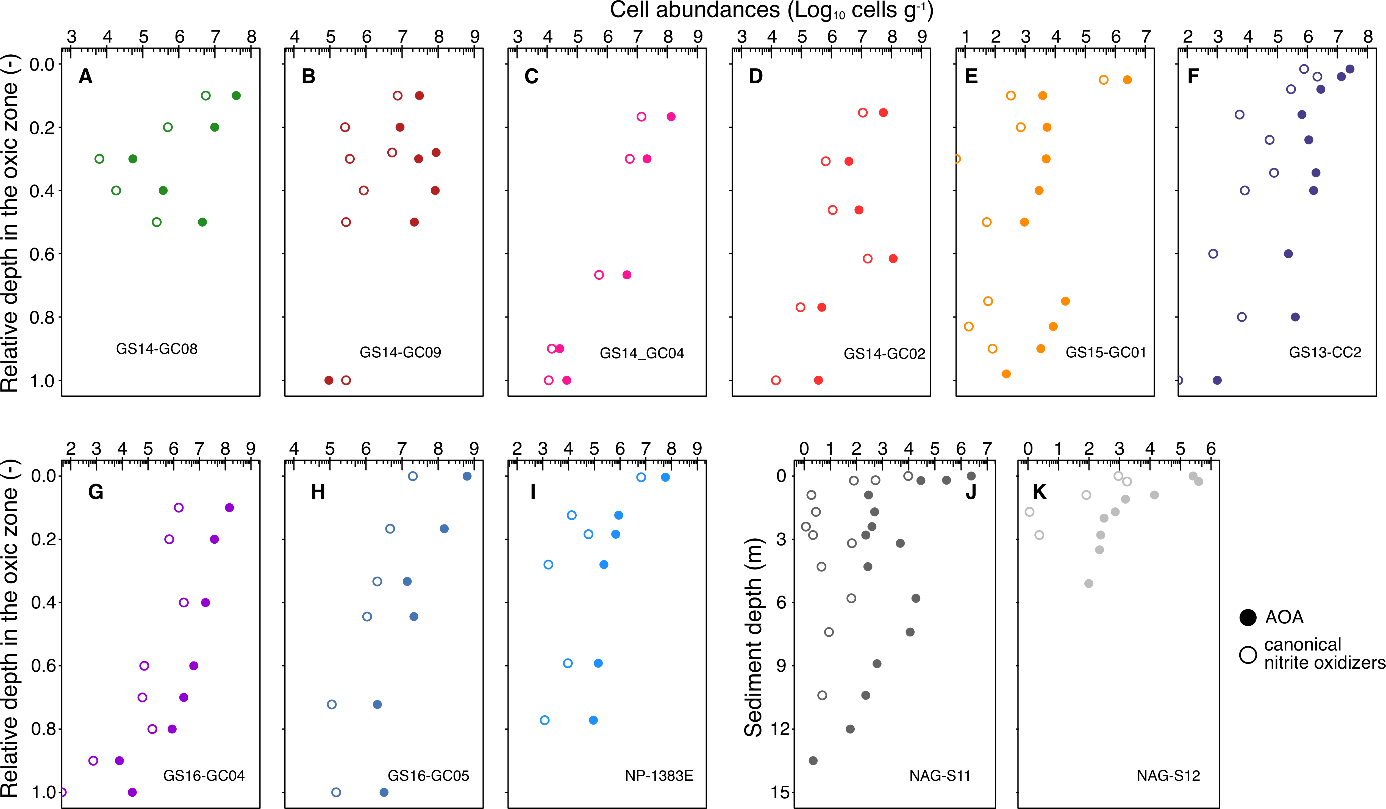
**

**Fig. S1. Absolute abundances of ammonia-oxidizing archaea (AOA) and canonical nitrite-oxidizing bacteria (the sum of *Nitrospiraceae* and *Nitrospinaceae*) in the oxic zones of eleven sediment cores with extensive oxic zones.** The abundances of AOA (filled circles) and canonical NOB (open circles) were calculated as the product of the total cell abundances and the relative abundances of the groups of interest in the total microbial communities as assessed by 16S rRNA gene amplicon sequencing. In cores **A**–**I**, relative depths within the oxic zone (0 = sediment surface, 1 = oxygen penetration depth) are shown to aid in comparison. Cores **J** and **K** are plotted against true depth below seafloor because the oxygen penetration depth was not resolved during sampling. Core numbers are indicated in each panel.


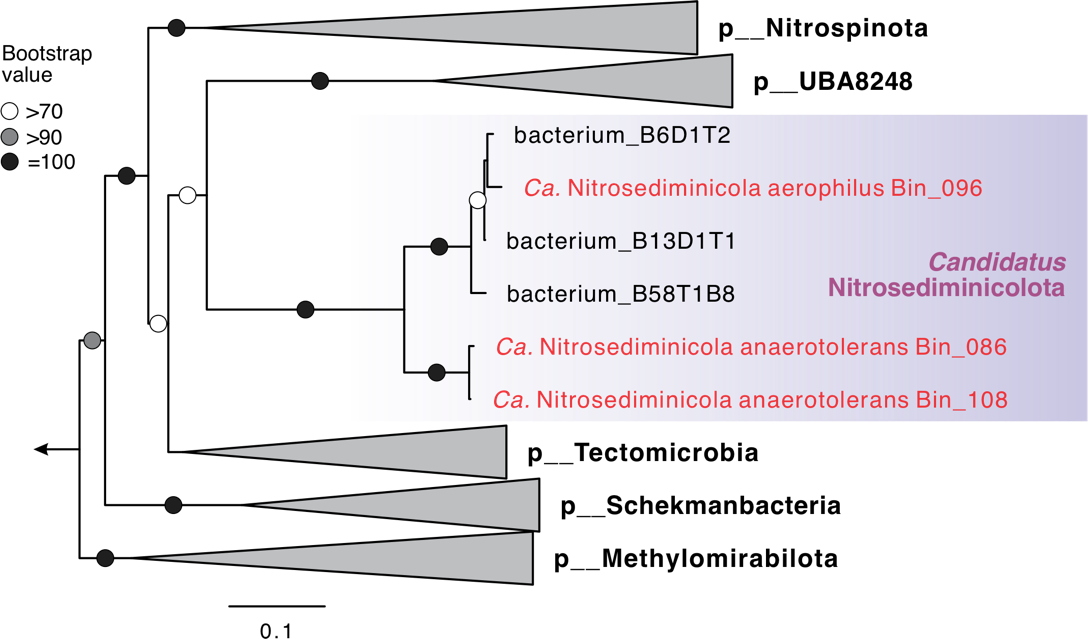


**Fig. S2. Maximum-likelihood phylogenetic tree of *Candidatus* Nitrosediminicolota and related bacterial phyla based on the concatenated 14 ribosomal proteins.** The tree is inferred using IQ-TREE with LG+R5 as the best-fit evolutionary model and 1,000 ultrafast bootstrap iterations. The nomenclature of the bacterial phyla follows GTDB, except that *Candidatus* Nitrosediminicolota is proposed in this study.


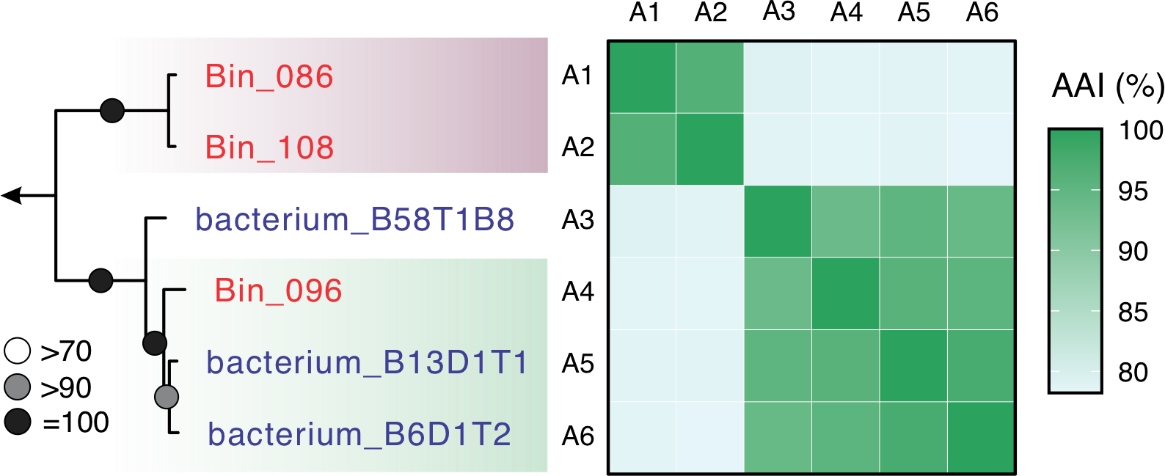


**Fig. S3. Average amino acid identity (AAI) between genomes in the newly proposed phylum *Candidatus* Nitrosediminicolota.** The three MAGs recovered in this study are highlighted in red, whereas those pre-existing from previous studies are in blue. The phylogenetic tree shown on the left is a subset of Fig. 2A. The lineage with the red background is *Ca.* N. anaerotolerans, while the one with light green background represents *Ca.* N. aerophilus.


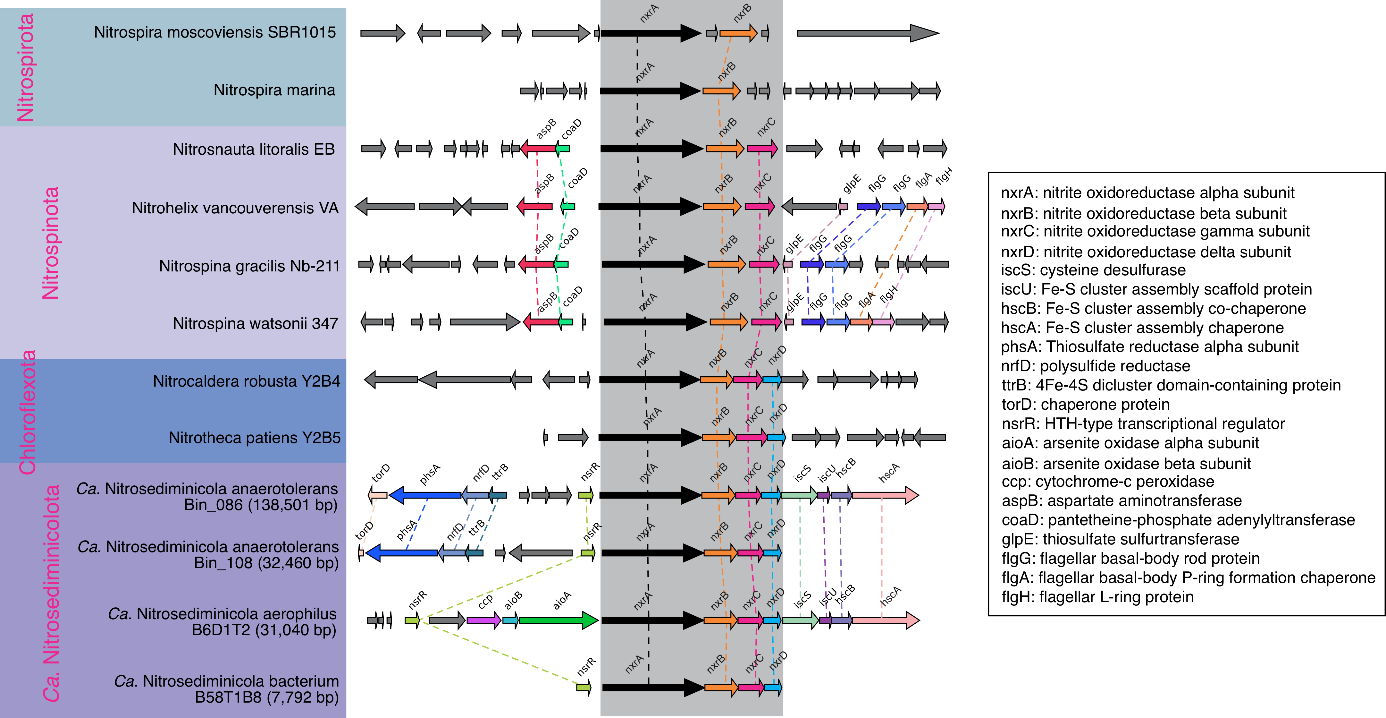


**Fig. S4. Arrangement of genes around the nitrite oxidoreductase (NXR) genes in selected nitrite-oxidizing bacteria.** While the paper focuses on *Ca.* Nitrosediminicola genomes, several selected NOB representatives from the Nitrospirota, Nitrospinota, *Nitrotoga*, and Chloroflexota taxa are also included for comparison. Homologs of NXR and other annotated enzymes are shown in various colors and connected with dashed lines, while the hypothetical genes are shown in grey. The annotated functions of these genes are shown on the right. The gene arrangements of NXR across the NOB genomes are highlighted by a grey box. For the *Ca.* Nitrosediminicola genomes, the lengths of the scaffolds in which the NXRs are located are also indicated in the parentheses after the genome names.


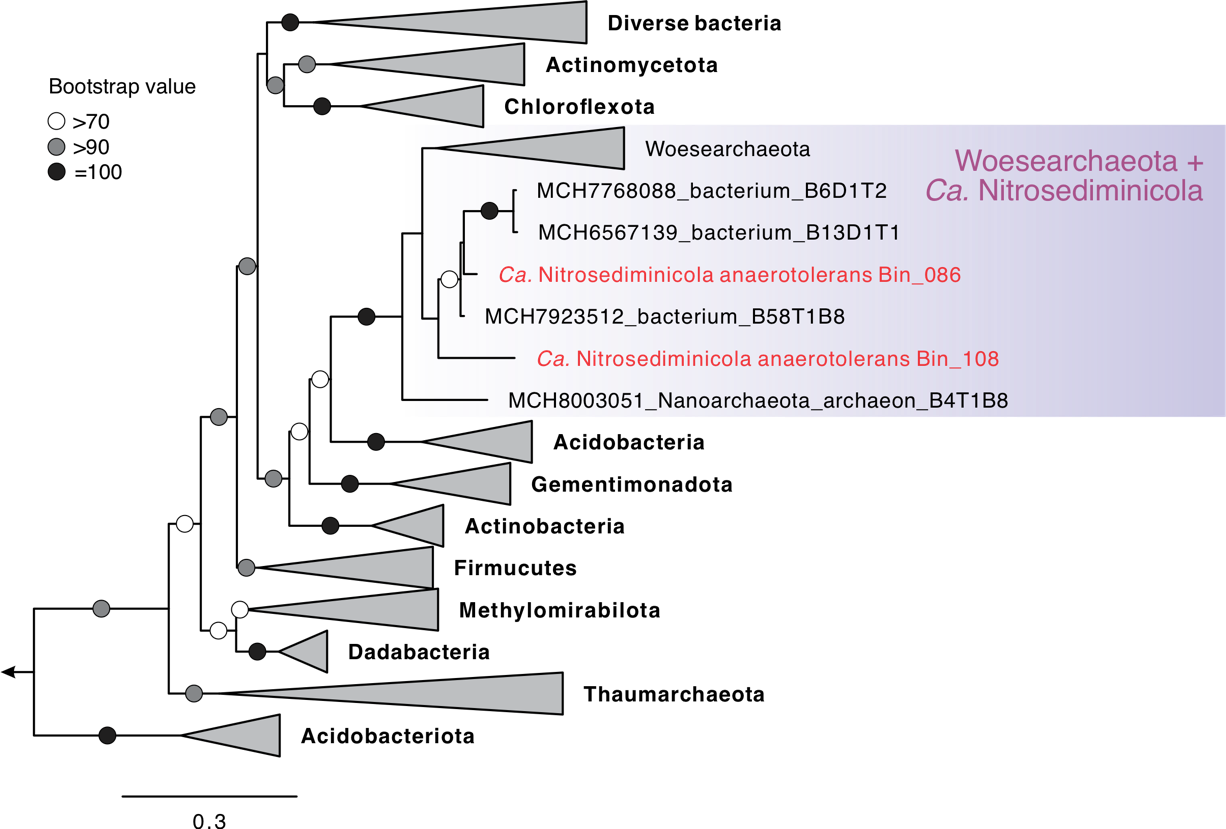


**Fig. S5. Maximum-likelihood phylogenetic tree of copper-containing nitrite reductase (NirK).** All sequences included here belong to “Clade II” of NirK. Only sequences of *Ca.* Nitrosediminicola described in this study are shown, while sequences of other various bacterial phyla are collapsed. The tight association of NirK sequences between *Ca.* Nitrosediminicola and the archaeal lineage *Ca.* Woesearchaeota is highlighted by a purple box.

**
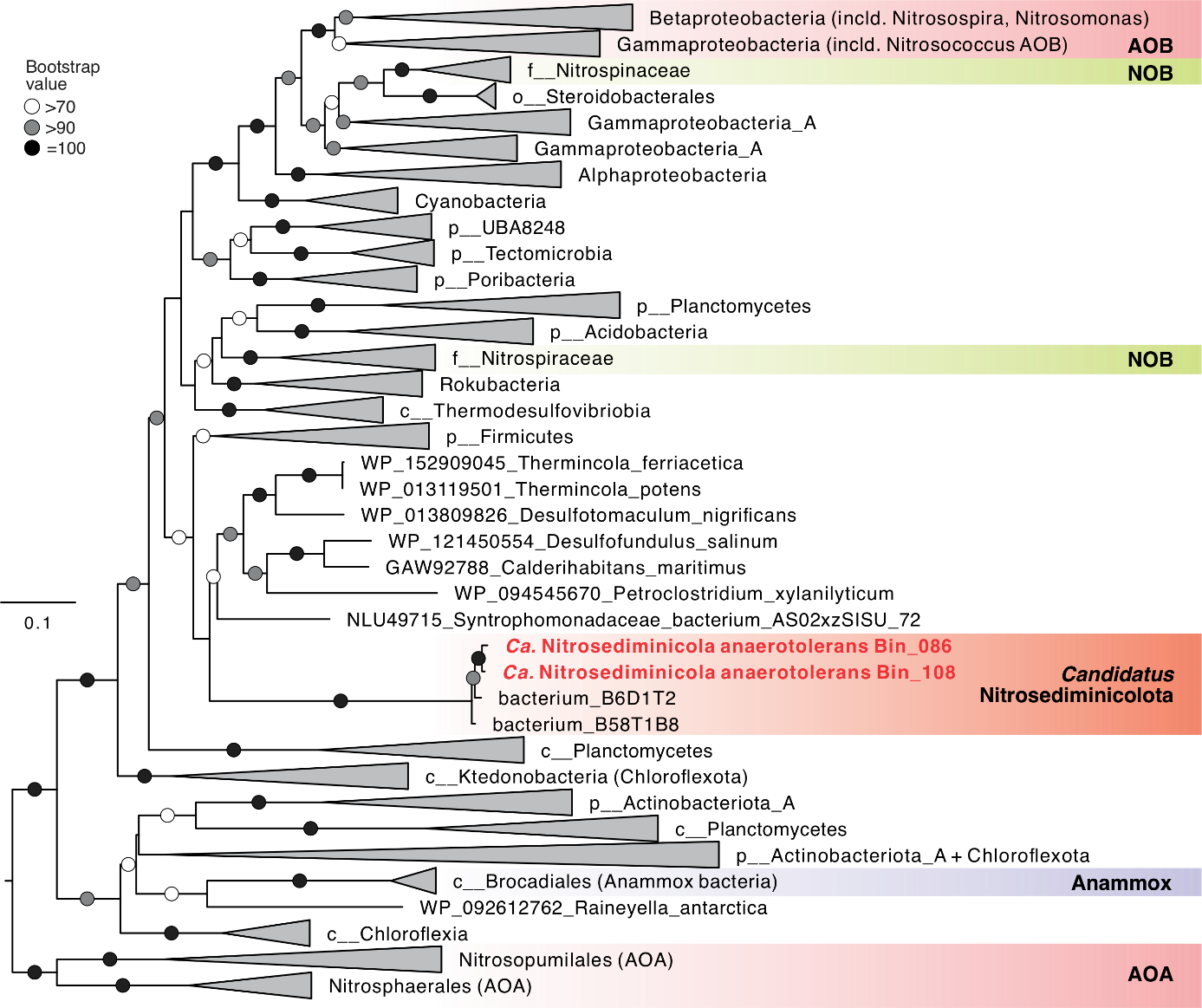
**

**Figure S6. Maximum-likelihood phylogenetic tree of UreC (urease alpha subunit).** The phylogeny was reconstructed using IQ-tree v1.6.10 under the LG+C20+F+G substitution model with 1,000 ultrafast bootstraps. The tree was rooted to NarG sequences of NC10 bacteria. *Ca.* Nitrosediminicolota genomes recovered in this study are shown in red. Bacteria known for having the capacity of nitrite oxidation, i.e., nitrite-oxidizing bacteria of the families of *Nitrospiraceae* and *Nitrospinaceae*, and anammox bacteria of the Brocadiales order are highlighted in green and purple boxes, respectively. AOA and AOB are highlighted in red boxes. Bootstrap values >70 are shown with symbols listed in the legend. The scale bar shows estimated sequence substitutions per residue.

**
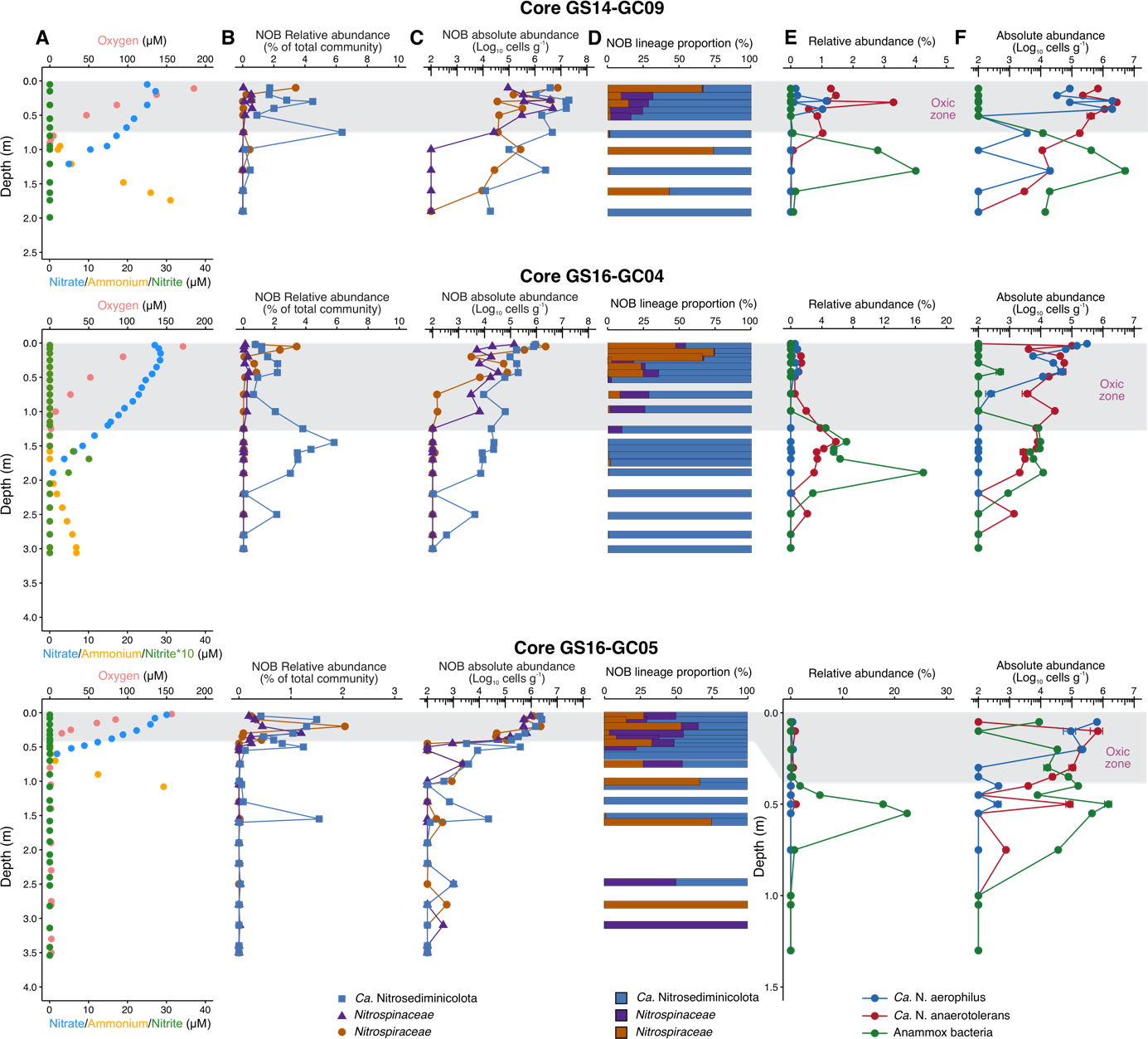
**

**Fig. S7. Geochemical context, relative abundances, and community compositions of NOB lineages in AMOR cores GS14-GC09, GS16-GC04, and GS16-GC05. (A)** Geochemical context delineated by the measured profiles of oxygen, nitrate, nitrite, and ammonium, previously reported in Zhao et al. (2020). The oxic zone in each core is marked with a grey box. **(B)** The relative abundances of *Ca.* Nitrosediminicolota and the canonical marine NOB families *Nitrospiraceae* and *Nitrospinaceae*, as assessed by amplicon sequencing. **(C)** The absolute abundances of the three NOB lineages calculated as the product of the relative abundances of the three lineages and the total cell numbers. (**D**) The community composition of NOB community in each of the investigated depth. (**E, F**) The relative (**E**) and absolute (**F**) abundances of two *Ca.* Nitrosediminicola species (*Ca.* N. aerophilus and *Ca.* N. anaerotolerans) and anammox bacteria throughout the core. Note the last two panels in core GS16-GC05 are shown in a small vertical scale to highlight the variations in the upper sediment layers.

**Supplemental references**

1. Pedersen H, Lomstein BA, Blackburn TH. Evidence for bacterial urea production in marine sediments. *FEMS Microbiology Ecology* **12**, 51-59 (1993).

2. Lomstein BA, Blackburn TH, Henriksen K. Aspects of nitrogen and carbon cycling in the northern Bering Shelf sediment. I. The significance of urea turnover in the mineralization of NH₄⁺. *Marine Ecology Progress Series*, 237-247 (1989).

3. Zhao R*, et al.* Geochemical transition zone powering microbial growth in subsurface sediments. *Proceedings of the National Academy of Sciences* **117**, 32617-32626 (2020).

4. Zhao R, Bauer SLM, Babbin AR. "Candidatus Subterrananammoxibiaceae", a new anammox bacterial family in globally distributed marine and terrestrial subsurfaces. *Applied and Environmental Microbiology* **89**, e00800-00823 (2023).

5. Kerou M*, et al.* Genomes of Thaumarchaeota from deep sea sediments reveal specific adaptations of three independently evolved lineages. *The ISME Journal* **15**, 2792-2808 (2021).

6. Koch H*, et al.* Expanded metabolic versatility of ubiquitous nitrite-oxidizing bacteria from the genus Nitrospira. *Proceedings of the National Academy of Sciences of the United States of America* **112**, 11371-11376 (2015).

7. Park S-J, Andrei A-Ş, Bulzu P-A, Kavagutti VS, Ghai R, Mosier AC. Expanded diversity and metabolic versatility of marine nitrite-oxidizing bacteria revealed by cultivation- and genomics-based approaches. *Applied and Environmental Microbiology* **86**, e01667-01620 (2020).

8. Mueller AJ*, et al.* Genomic and kinetic analysis of novel Nitrospinae enriched by cell sorting. *The ISME Journal* **15**, 732-745 (2021).

9. Luecker S, Nowka B, Rattei T, Spieck E, Daims H. The genome of Nitrospina gracilis illuminates the metabolism and evolution of the major marine nitrite oxidizer. *Frontiers in Microbiology* **4**, 27 (2013).

10. Ngugi DK, Blom J, Stepanauskas R, Stingl U. Diversification and niche adaptations of Nitrospina-like bacteria in the polyextreme interfaces of Red Sea brines. *The ISME Journal* **10**, 1383-1399 (2016).

11. Lawson CE*, et al.* Investigating the chemolithoautotrophic and formate metabolism of Nitrospira moscoviensis by constraint-based metabolic modeling and 13C-tracer analysis. *mSystems* **6**, e00173-00121 (2021).

12. Bayer B*, et al.* Metabolic versatility of the nitrite-oxidizing bacterium Nitrospira marina and its proteomic response to oxygen-limited conditions. *The ISME Journal* **15**, 1025-1039 (2021).

13. Kitzinger K*, et al.* Characterization of the first "Candidatus Nitrotoga" isolate reveals metabolic versatility and separate evolution of widespread nitrite-oxidizing bacteria. *mBio* **9**, e01186-01118 (2018).
