# Supplementary material for: An abundant bacterial phylum with nitrite-oxidizing potential in oligotrophic marine sediments
Source: Commun Biol. 2024 Apr 11;7:449. doi: 10.1038/s42003-024-06136-2 (PMC11009272; doi:10.1038/s42003-024-06136-2)
Supplement: Supplementary file 2 — Description of Additional Supplementary Files [file 42003_2024_6136_MOESM2_ESM.pdf]

Description of Additional Supplementary Files

File name: Supplementary Data 1
Description: Relative and absolute abundances of AOA and NOB in a total of 17 sediment cores. It contains the underlying data for Figure 1.

File name: Supplementary Data 2
Description: Relative abundance, habitat type, and coordinates of samples containing >0.1% of Ca. Nitrosediminicolota bacteria. It contains the underlying data for Figure 2D.

File name: Supplementary Data 3
Description: Relative abundances of three lineages of NOB (Ca. Nitrosediminicolota and the canonical Nitrospiraceae and Nitrospinaceae) and also anammox bacteria and the geochemical profiles in four Arctic sediment cores. It contains the underlying data for Figure 5.

File name: Supplementary Data 4
Description: Comparative genomic analysis showing the unique gene clusters in Ca. N. aerophilus and Ca. N. anaerotolerans.
