# Supplementary material for: An abundant bacterial phylum with nitrite-oxidizing potential in oligotrophic marine sediments
Source: Commun Biol. 2024 Apr 11;7:449. doi: 10.1038/s42003-024-06136-2 (PMC11009272; doi:10.1038/s42003-024-06136-2)
Supplement: Supplementary file 7 — Reporting Summary [file 42003_2024_6136_MOESM7_ESM.pdf]

nature portfolio

Corresponding author(s): Rui Zhao, Andrew R. Babbin

Last updated by author(s): Mar 22, 2024

Reporting Summary

Nature Portfolio wishes to improve the reproducibility of the work that we publish. This form provides structure for consistency and transparency in reporting. For further information on Nature Portfolio policies, see our Editorial Policies and the Editorial Policy Checklist.

Statistics

For all statistical analyses, confirm that the following items are present in the figure legend, table legend, main text, or Methods section.

n/a	Confirmed	
☐	☒	The exact sample size (*n*) for each experimental group/condition, given as a discrete number and unit of measurement
☐	☒	A statement on whether measurements were taken from distinct samples or whether the same sample was measured repeatedly
☐	☒	The statistical test(s) used AND whether they are one- or two-sided<br>*Only common tests should be described solely by name; describe more complex techniques in the Methods section.*
☒	☐	A description of all covariates tested
☒	☐	A description of any assumptions or corrections, such as tests of normality and adjustment for multiple comparisons
☐	☒	A full description of the statistical parameters including central tendency (e.g. means) or other basic estimates (e.g. regression coefficient) AND variation (e.g. standard deviation) or associated estimates of uncertainty (e.g. confidence intervals)
☒	☐	For null hypothesis testing, the test statistic (e.g. *F*, *t*, *r*) with confidence intervals, effect sizes, degrees of freedom and *P* value noted<br>*Give P values as exact values whenever suitable.*
☒	☐	For Bayesian analysis, information on the choice of priors and Markov chain Monte Carlo settings
☒	☐	For hierarchical and complex designs, identification of the appropriate level for tests and full reporting of outcomes
☒	☐	Estimates of effect sizes (e.g. Cohen's *d*, Pearson's *r*), indicating how they were calculated

Our web collection on statistics for biologists contains articles on many of the points above.

Software and code

Policy information about availability of computer code

Data collection	No software was used.
Data analysis	USEARCH v11.0.667 (amplicon sequencing data processing, including read quality filtering, merge, OTU clustering),<br>R package pracma (depth-integration relative abundance calculation),<br>MEGAHIT v1.1.2 (metagenome reads assembly),<br>CheckM2 v1.0.2 (genome bin quality assessment),<br>SPAdes v3.12.0 (genome re-assembly),<br>gbtools v2.6.0 (genome visualization and manual binning),<br>GTDB-tk v2.3.0 (microbial genome classification),<br>Prokka v1.13 (bacterial genome annotation),<br>Anvi’o v7.1 (comparative genome analysis),<br>IQ-TREE v1.5.5 (phylogenetic tree reconstruction).

For manuscripts utilizing custom algorithms or software that are central to the research but not yet described in published literature, software must be made available to editors and reviewers. We strongly encourage code deposition in a community repository (e.g. GitHub). See the Nature Portfolio guidelines for submitting code & software for further information.

Data

Policy information about availability of data

All manuscripts must include a data availability statement. This statement should provide the following information, where applicable:

- Accession codes, unique identifiers, or web links for publicly available datasets
- A description of any restrictions on data availability
- For clinical datasets or third party data, please ensure that the statement adheres to our policy

All sequencing data used in this study are available in the NCBI Short Reads Archive under the project number PRJNA529480. The three Ca. Nitrosediminicolota genomes recovered in this study are available under the accession number JAWJBM000000000 (Bin_086), JAWJBN000000000 (Bin_108), and JAWJBO000000000 (Bin_096).

Research involving human participants, their data, or biological material

Policy information about studies with human participants or human data. See also policy information about sex, gender (identity/presentation), and sexual orientation and race, ethnicity and racism.

Reporting on sex and gender	not relevant
Reporting on race, ethnicity, or other socially relevant groupings	not relevant
Population characteristics	not relevant
Recruitment	not relevant
Ethics oversight	not relevant

Note that full information on the approval of the study protocol must also be provided in the manuscript.

Field-specific reporting

Please select the one below that is the best fit for your research. If you are not sure, read the appropriate sections before making your selection.

☐ Life sciences ☐ Behavioural & social sciences ☒ Ecological, evolutionary & environmental sciences

For a reference copy of the document with all sections, see nature.com/documents/nr-reporting-summary-flat.pdf

Ecological, evolutionary & environmental sciences study design

All studies must disclose on these points even when the disclosure is negative.

Study description	We identified an apparent abundance mismatch between two groups of nitrifying organisms in marine sediments and addressed this question by discovering a new abundant phylum of bacteria that putatively perform nitrite oxidation.
Research sample	All research samples are marine sediments retrieved from the seabed of the Arctic and Pacific Ocean.
Sampling strategy	We included all oxic sediment samples for which oxygen concentration and microbiome sequencing data are available.
Data collection	Oxygen concentration data were collected during the oceanographic cruises. Other data were measured and analyzed in the laboratories of the authors.
Timing and spatial scale	not relevant
Data exclusions	No data were excluded.
Reproducibility	All attempts to repeat the analyses were successful.
Randomization	Not relevant, because we analyzed all samples collected.
Blinding	Sampling and analyses were conducted by different people in the author team

Did the study involve field work? ☒ Yes ☐ No

Field work, collection and transport

Field conditions	Seafloor beneath the Norwegian and Greenland Seas
Location	Seafloor of 1500-2500 meters below the sea level.
Access & import/export	Access the sampling sites via research vessel G.O. Sars of Norway. No permit is required to sample the seabed of the sampling area.
Disturbance	No disturbance

Reporting for specific materials, systems and methods

We require information from authors about some types of materials, experimental systems and methods used in many studies. Here, indicate whether each material, system or method listed is relevant to your study. If you are not sure if a list item applies to your research, read the appropriate section before selecting a response.

Materials & experimental systems

n/a	Involved in the study
☒	☐ Antibodies
☒	☐ Eukaryotic cell lines
☒	☐ Palaeontology and archaeology
☒	☐ Animals and other organisms
☒	☐ Clinical data
☒	☐ Dual use research of concern
☒	☐ Plants

Methods

n/a	Involved in the study
☒	☐ ChIP-seq
☒	☐ Flow cytometry
☒	☐ MRI-based neuroimaging

Plants

Seed stocks	not relevant
Novel plant genotypes	not relevant
Authentication	not relevant
